# Supplementary material for: Global transboundary synergies and trade-offs among Sustainable Development Goals from an integrated sustainability perspective
Source: Nat Commun. 2024 Jan 13;15:500. doi: 10.1038/s41467-023-44679-w (PMC10786910; doi:10.1038/s41467-023-44679-w)
Supplement: Supplementary file 1 — Supplementary information [file 41467_2023_44679_MOESM1_ESM.pdf]

Supplementary Information for:

Global transboundary synergies and trade-offs among Sustainable Development Goals from an integrated sustainability perspective

Huijuan Xiao[1,2], Sheng Bao[3], Jingzheng Ren[2,4,5,*], Zhenci Xu[6,7,*], Song Xue[2], Jianguo Liu[8,*]

[1] Department of Civil and Environmental Engineering, The Hong Kong University of Science and Technology, Hong Kong SAR, China

[2] Department of Industrial and Systems Engineering, The Hong Kong Polytechnic University, Hong Kong SAR, China

[3] Otto Poon C. F. Smart Cities Research Institute, Department of Land Surveying and Geo-Informatics, The Hong Kong Polytechnic University, Hong Kong SAR, China

[4] Research Center for Resources Engineering Towards Carbon Neutrality, The Hong Kong Polytechnic University, Hong Kong SAR, China

[5] Department of Industrial and Systems Engineering, Research Institute for Advanced Manufacturing, The Hong Kong Polytechnic University, Hong Kong SAR, China

[6] Department of Geography, The University of Hong Kong, Hong Kong SAR, China

[7] Shenzhen Institute of Research and Innovation, The University of Hong Kong, Hong Kong SAR, China

[8] Center for Systems Integration and Sustainability, Department of Fisheries and Wildlife, Michigan State University, East Lansing, MI, 48824, United States of America

Corresponding Addresses:

liuji@msu.edu (J. L.); jzhren@polyu.edu.hk (J.R.); xuzhenci@hku.hk (Z. X.)

Table of contents

Table S1. SDG indicator list. 2

Table S2. Causal relationship across 55 SDG indicators. 4

Table S3. Country list.. 7

Table S4. Country classification by income level. 8

Table S5. Variable selection and data sources. 11

Table S6. Explanatory variables and selection rationale for 55 SDG indicators. 12

Table S7. Coastal country list. 50

Table S8. Magnitude of transboundary interactions. 52

Table S1. SDG indicator list. 55 SDG indicators covering all 17 SDGs were included in analysis.

No.	SDG target	SDG indicator
1	1.4	Proportion of population using basic drinking water services, by location (%)
2	1.4	Proportion of population using basic sanitation services, by location (%)
3	2.2	Proportion of women aged 15-49 years with anaemia, non-pregnant (%)
4	2.2	Proportion of women aged 15-49 years with anaemia, pregnant (%)
5	2.3	Cereal yield (kg per hectare)
6	3.1	Maternal mortality ratio
7	3.2	Infant mortality rate (deaths per 1,000 live births)
8	3.2	Neonatal mortality rate (deaths per 1,000 live births)
9	3.3	Tuberculosis incidence (per 100,000 population)
10	4.1	Primary education, duration (years)
11	4.2	School enrollment, preprimary (% gross)
12	4.5	School enrollment, primary (gross), gender parity index (GPI)
13	5.1	Women Business and the Law Index Score (scale 1-100)
14	5.4	Contributing family workers, female (% of female employment) (modeled ILO estimate)
15	5.5	Proportion of seats held by women in national parliaments (% of total number of seats)
16	6.2	Proportion of population practicing open defecation, by urban/rural (%)
17	6.4	Water Use Efficiency (United States dollars per cubic meter)
18	6.4	Level of water stress: freshwater withdrawal as a proportion of available freshwater resources (%)
19	6.6	Lakes and rivers permanent water area (% of total land area)
20	6.6	Lakes and rivers seasonal water area (% of total land area)
21	7.1	Proportion of population with access to electricity, by urban/rural (%)
22	7.1	Proportion of population with primary reliance on clean fuels and technology (%)
23	7.2	Renewable energy share in the total final energy consumption (%)
24	7.3	Energy intensity level of primary energy (megajoules per constant 2017 purchasing power parity GDP)
25	8.1	Number of commercial bank branches per 100,000 adults
26	8.4	Domestic material consumption per unit of GDP, by type of raw material (kilograms per constant 2015 United States dollars)
27	8.4	Domestic material consumption per capita, by type of raw material (tonnes)

28	8.5	Wage and salaried workers, total (% of total employment) (modeled ILO estimate)
29	9.2	Manufacturing value added (constant 2015 United States dollars) as a proportion of GDP (%)
30	10.4	Labour share of GDP (%)
31	10.7	Number of refugees per 100,000 population, by country of origin (per 100,000 population)
32	11.1	Urban population (% of total population)
33	11.1	Urban population growth (annual %)
34	11.6	Annual mean levels of fine particulate matter (population-weighted), by location (micrograms per cubic meter)
35	11.6	PM2.5 air pollution, population exposed to levels exceeding WHO guideline value (% of total)
36	11.6	PM2.5 air pollution, mean annual exposure (micrograms per cubic meter)
37	12.2	Total natural resources rents (% of GDP)
38	12.2	Domestic material consumption per unit of GDP, by type of raw material (kilograms per constant 2015 United States dollars)
39	12.2	Domestic material consumption per capita, by type of raw material (tonnes)
40	13.1	Total number of people affected by floods per 100,000
41	13.1	Energy-related CO_2 emissions per capita (tCO_2/capita)
42	14.4	Anual growth rate of capture fisheries production
43	14.4	Annual growth rate of aquaculture production (of total %)
44	14.4	Annual growth rate of total fisheries production (metric tons)
45	15.1	Forest area (% of land area)
46	15.1	Average proportion of Terrestrial Key Biodiversity Areas (KBAs) covered by protected areas (%)
47	15.4	Average proportion of Mountain Key Biodiversity Areas (KBAs) covered by protected areas (%)
48	15.5	Red List Index
49	16.1	Annual number of deaths from homicide per 100,000 people
50	16.1	homicide rate by age (15-49)
51	17.3	Volume of remittances (in United States dollars) as a proportion of total GDP (%)
52	17.6	Fixed Internet broadband subscriptions per 100 inhabitants, by speed (per 100 inhabitants)
53	17.8	Internet users per 100 inhabitants
54	17.12	Average tariff applied by developed countries, most-favored nation status, by type of product (%)
55	17.13	Foreign direct investment, net inflows, as a proportion of GDP (%)

Table S2. Causal relationship across 55 SDG indicators. The number under interaction generator represents corresponding SDG indicator. For example, interaction generator 1 indicates 'Proportion of population using basic drinking water services, by location (%)' and it can have interactions with 24 SDG indicators (1;3;4;5;16;19;20;21;22;23;24;25;28;29;32;33;37;38;39;42;43;44;45;46). Based on 55 SDG indicators, we identified a total of 768 SDG indicator pairs with the causal relationships, which can be derived from a database named interactive repository of SDG interactions in CDEdatablog[3].

No.	Interaction receiver	Interaction generator
1	Proportion of population using basic drinking water services, by location (%)	1;3;4;5;16;19;20;21;22;23;24;25;28;29;32;33;37;38;39;42;43;44;45;46
2	Proportion of population using basic sanitation services, by location (%)	2;3;4;5;16;19;20;21;22;23;24;25;28;29;32;33;37;38;39;42;43;44;45;46
3	Proportion of women aged 15-49 years with anaemia, non-pregnant (%)	3;7;8;10;13;14;15;16;42;43;44;45;46
4	Proportion of women aged 15-49 years with anaemia, pregnant (%)	4;7;8;10;13;14;15;16;42;43;44;45;46
5	Cereal yield (kg per hectare)	5;1;2;9;19;20;21;22;23;24;40;41;45;46;47;48
6	Maternal mortality ratio	6;3;4;5;9;16;21;22;23;28
7	Infant mortality rate (deaths per 1,000 live births)	7;3;4;5;9;12;16;21;22;23;24;28;32;33
8	Neonatal mortality rate (deaths per 1,000 live births)	8;3;4;5;9;12;16;21;22;23;24;28;32;33
9	Tuberculosis incidence (per 100,000 population)	9;3;4;5;10;16;21;22;23;25;28;31;32;33;40;41;48
10	Primary education, duration (years)	10;11;16;21;22;24;25;42;43;44;49;50
11	School enrollment, preprimary (% gross)	11;16;21;22;24
12	School enrollment, primary (gross), gender parity index (GPI)	12;16;21;22;24
13	Women Business and the Law Index Score (scale 1-100)	13;3;4;16;21;22;23;24;40;41
14	Contributing family workers, female (% of female employment) (modeled ILO estimate)	14;3;4;16;21;22;23
15	Proportion of seats held by women in national parliaments (% of total number of seats)	15;1;2;3;4;5;13;16;21;22;24;49;50
16	Proportion of population practicing open defecation, by urban/rural (%)	16;5;15;21;22;23;24;26;27;34;35;36;37;38;39
17	Water Use Efficiency (United States dollars per cubic meter)	17;1;2;3;4;5;21;22;23;24;25;26;27;32;33;34;35;36;37;38;39
18	Level of water stress: freshwater withdrawal as a proportion of available freshwater resources (%)	18;1;2;3;4;5;21;22;23;24;25;26;27;32;33;34;35;36;37;38;39

19	Lakes and rivers permanent water area (% of total land area)	19;1;2;3;4;5;21;22;23;24;25;26;27;32;33;34;35;36;37;38;39;45;46;49;50
20	Lakes and rivers seasonal water area (% of total land area)	20;1;2;3;4;5;21;22;23;24;25;26;27;32;33;34;35;36;37;38;39;45;46;49;50
21	Proportion of population with access to electricity, by urban/rural (%)	21;3;4;5;17;18;23;24;32;33;34;35;36;45;46;48;49;50;52;53
22	Proportion of population with primary reliance on clean fuels and technology (%)	22;3;4;5;17;18;23;24;32;33;34;35;36;45;46;48;49;50;52;53
23	Renewable energy share in the total final energy consumption (%)	23;3;4;5;16;17;18;19;20;24;25;28;37;38;39;45;46;47;48
24	Energy intensity level of primary energy (megajoules per constant 2017 purchasing power parity GDP)	24;17;18;19;20;23;25;28;37;38;39;45;46;49;50;52
25	Number of commercial bank branches per 100,000 adults	25;5;6;7;8;9;10;16;17;18;19;20;21;22;23;24;28;29;37;38;39;42;43;44;45;46;47;49;50
26	Domestic material consumption per unit of GDP, by type of raw material (kilograms per constant 2015 United States dollars)	26;1;2;16;17;18;19;20;21;22;23;24;37;38;39;42;43;44
27	Domestic material consumption per capita, by type of raw material (tonnes)	27;1;2;16;17;18;19;20;21;22;23;24;37;38;39;42;43;44
28	Wage and salaried workers, total (% of total employment) (modeled ILO estimate)	28;5;6;7;8;9;11;16;21;22;23;24;28;29;31;34;35;36;37;38;39;42;43;44;45;46
29	Manufacturing value added (constant 2015 United States dollars) as a proportion of GDP (%)	29;17;18;19;20;37;38;39
30	Labour share of GDP (%)	30;21;22;23;24
31	Number of refugees per 100,000 population, by country of origin (per 100,000 population)	31;40;41
32	Urban population (% of total population)	32;21;22;24;48
33	Urban population growth (annual %)	33;21;22;24;48
34	Annual mean levels of fine particulate matter (population-weighted), by location (micrograms per cubic meter)	34;16;17;18;19;20;21;22;23;24;45;46
35	PM2.5 air pollution, population exposed to levels exceeding WHO guideline value (% of total)	35;16;17;18;19;20;21;22;23;24;45;46
36	PM2.5 air pollution, mean annual exposure (micrograms per cubic meter)	36;16;17;18;19;20;21;22;23;24;45;46
37	Total natural resources rents (% of GDP)	37;16;17;18;19;20;21;22;23;24;26;27;28;42;43;44
38	Domestic material consumption per unit of GDP, by type of raw material (kilograms per constant 2015 United States dollars)	38;16;17;18;19;20;21;22;23;24;26;27;28;42;43;44
39	Domestic material consumption per capita, by type of raw material (tonnes)	39;16;17;18;19;20;21;22;23;24;26;27;28;42;43;44

40	Total number of people affected by floods per 100,000	40;5;10;19;20;21;22;23;24;25;32;33;37;38;39;42;43;44;45;46;48;49;50
41	Energy-related CO2 emissions per capita (tCO2/capita)	41;5;10;19;20;21;22;23;24;25;32;33;37;38;39;42;43;44;45;46;48;49;50
42	Anual growth rate of capture fisheries production	42;23;25;28
43	Annual growth rate of aquaculture production (of total %)	43;23;25;28
44	Annual growth rate of total fisheries production (metric tons)	44;23;25;28
45	Forest area (% of land area)	45;1;2;3;4;5;17;18;19;20;21;22;23;26;27;28;32;33;34;35;36;37;38;39;47;48;49;50
46	Average proportion of Terrestrial Key Biodiversity Areas (KBAs) covered by protected areas (%)	46;1;2;3;4;5;17;18;19;20;21;22;23;26;27;28;32;33;34;35;36;37;38;39;47;48;49;50
47	Average proportion of Mountain Key Biodiversity Areas (KBAs) covered by protected areas (%)	47;21;22;23;45;46;48
48	Red List Index	48;19;20;21;22;23;26;27;34;35;36;37;38;39;45;46
49	Annual number of deaths from homicide per 100,000 people	49;1;2;23;28;37;38;39
50	homicide rate by age(15-49)	50;1;2;23;28;37;38;39
51	Volume of remittances (in United States dollars) as a proportion of total GDP (%)	51;10;11;12;30;49;50
52	Fixed Internet broadband subscriptions per 100 inhabitants, by speed (per 100 inhabitants)	52;10;11;12;30;49;50
53	Internet users per 100 inhabitants	53;10;11;12;30;49;50
54	Average tariff applied by developed countries, most-favored nation status, by type of product (%)	54;10;11;12;30;49;50
55	Foreign direct investment, net inflows, as a proportion of GDP (%)	55;10;11;12;30;49;50

Table S3. Country list. 121 countries are included in analysis.

No	Code	Country	No	Code	Country
1	AFG	Afghanistan	62	JAM	Jamaica
2	AGO	Angola	63	JOR	Jordan
3	ALB	Albania	64	KAZ	Kazakhstan
4	ARG	Argentina	65	KEN	Kenya
5	ARM	Armenia	66	KGZ	Kyrgyz Republic
6	AUS	Australia	67	KHM	Cambodia
7	AUT	Austria	68	KOR	Korea, Rep.
8	AZE	Azerbaijan	69	LAO	Lao PDR
9	BDI	Burundi	70	LSO	Lesotho
10	BEL	Belgium	71	LTU	Lithuania
11	BEN	Benin	72	LVA	Latvia
12	BFA	Burkina Faso	73	MAR	Morocco
13	BGD	Bangladesh	74	MDA	Moldova
14	BLZ	Belize	75	MDG	Madagascar
15	BOL	Bolivia	76	MEX	Mexico
16	BRA	Brazil	77	MLI	Mali
17	BRB	Barbados	78	MMR	Myanmar
18	BTN	Bhutan	79	MUS	Mauritius
19	BWA	Botswana	80	MWI	Malawi
20	CAF	Central African Republic	81	MYS	Malaysia
21	CAN	Canada	82	NAM	Namibia
22	CHL	Chile	83	NER	Niger
23	CHN	China	84	NGA	Nigeria
24	CIV	Ivory Coast	85	NIC	Nicaragua
25	CMR	Cameroon	86	NOR	Norway
26	COD	Congo, Dem. Rep.	87	NPL	Nepal
27	COL	Colombia	88	NZL	New Zealand
28	CPV	Cabo Verde	89	OMN	Oman
29	CRI	Costa Rica	90	PAK	Pakistan
30	CYP	Cyprus	91	PAN	Panama
31	DEU	Germany	92	PER	Peru
32	DNK	Denmark	93	PHL	Philippines
33	DOM	Dominican Republic	94	PNG	Papua New Guinea
34	DZA	Algeria	95	PRT	Portugal
35	ECU	Ecuador	96	PRY	Paraguay
36	EGY	Egypt, Arab Rep.	97	QAT	Qatar
37	ESP	Spain	98	ROU	Romania
38	EST	Estonia	99	RUS	Russian Federation
39	ETH	Ethiopia	100	RWA	Rwanda
40	FIN	Finland	101	SAU	Saudi Arabia
41	FJI	Fiji	102	SLV	El Salvador
42	FRA	France	103	SUR	Suriname
43	GAB	Gabon	104	SVK	Slovak Republic
44	GBR	United Kingdom	105	SVN	Slovenia
45	GEO	Georgia	106	SWZ	Eswatini
46	GHA	Ghana	107	SYR	Syrian Arab Republic
47	GIN	Guinea	108	TGO	Togo
48	GMB	Gambia, The	109	TJK	Tajikistan

49	GRC	Greece	110	TTO	Trinidad and Tobago
50	GTM	Guatemala	111	TUN	Tunisia
51	HND	Honduras	112	TZA	Tanzania
52	HRV	Croatia	113	UGA	Uganda
53	HUN	Hungary	114	UKR	Ukraine
54	IDN	Indonesia	115	URY	Uruguay
55	IND	India	116	USA	United States
56	IRL	Ireland	117	UZB	Uzbekistan
57	IRN	Iran, Islamic Rep.	118	VNM	Vietnam
58	IRQ	Iraq	119	ZAF	South Africa
59	ISL	Iceland	120	ZMB	Zambia
60	ISR	Israel	121	ZWE	Zimbabwe
61	ITA	Italy			

Table S4. Country classification by income level.

No	Code	Group	No	Code	Group
1	AFG	Low Income	62	JAM	Upper Middle Income
2	AGO	Lower Middle Income	63	JOR	Upper Middle Income
3	ALB	Upper Middle Income	64	KAZ	Upper Middle Income
4	ARG	Upper Middle Income	65	KEN	Lower Middle Income
5	ARM	Upper Middle Income	66	KGZ	Lower Middle Income
6	AUS	High Income	67	KHM	Lower Middle Income
7	AUT	High Income	68	KOR	High Income
8	AZE	Upper Middle Income	69	LAO	Lower Middle Income
9	BDI	Low Income	70	LSO	Lower Middle Income
10	BEL	High Income	71	LTU	High Income
11	BEN	Lower Middle Income	72	LVA	High Income
12	BFA	Low Income	73	MAR	Lower Middle Income
13	BGD	Lower Middle Income	74	MDA	Upper Middle Income
14	BLZ	Upper Middle Income	75	MDG	Low Income
15	BOL	Lower Middle Income	76	MEX	Upper Middle Income
16	BRA	Upper Middle Income	77	MLI	Low Income
17	BRB	High Income	78	MMR	Lower Middle Income
18	BTN	Lower Middle Income	79	MUS	Upper Middle Income
19	BWA	Upper Middle Income	80	MWI	Low Income
20	CAF	Low Income	81	MYS	Upper Middle

					Income
21	CAN	High Income	82	NAM	Upper Middle Income
22	CHL	High Income	83	NER	Low Income
23	CHN	Upper Middle Income	84	NGA	Lower Middle Income
24	CIV	Lower Middle Income	85	NIC	Lower Middle Income
25	CMR	Lower Middle Income	86	NOR	High Income
26	COD	Low Income	87	NPL	Lower Middle Income
27	COL	Upper Middle Income	88	NZL	High Income
28	CPV	Lower Middle Income	89	OMN	High Income
29	CRI	Upper Middle Income	90	PAK	Lower Middle Income
30	CYP	High Income	91	PAN	High Income
31	DEU	High Income	92	PER	Upper Middle Income
32	DNK	High Income	93	PHL	Lower Middle Income
33	DOM	Upper Middle Income	94	PNG	Lower Middle Income
34	DZA	Lower Middle Income	95	PRT	High Income
35	ECU	Upper Middle Income	96	PRY	Upper Middle Income
36	EGY	Lower Middle Income	97	QAT	High Income
37	ESP	High Income	98	ROU	High Income
38	EST	High Income	99	RUS	Upper Middle Income
39	ETH	Low Income	100	RWA	Low Income
40	FIN	High Income	101	SAU	High Income
41	FJI	Upper Middle Income	102	SLV	Lower Middle Income
42	FRA	High Income	103	SUR	Upper Middle Income
43	GAB	Upper Middle Income	104	SVK	High Income
44	GBR	High Income	105	SVN	High Income
45	GEO	Upper Middle Income	106	SWZ	Lower Middle Income
46	GHA	Lower Middle Income	107	SYR	Low Income
47	GIN	Low Income	108	TGO	Low Income
48	GMB	Low Income	109	TJK	Lower Middle Income
49	GRC	High Income	110	TTO	High Income
50	GTM	Upper Middle Income	111	TUN	Lower Middle Income
51	HND	Lower Middle Income	112	TZA	Lower Middle Income
52	HRV	High Income	113	UGA	Low Income
53	HUN	High Income	114	UKR	Lower Middle Income
54	IDN	Lower Middle Income	115	URY	High Income
55	IND	Lower Middle Income	116	USA	High Income

56	IRL	High Income	117	UZB	Lower Middle Income
57	IRN	Lower Middle Income	118	VNM	Lower Middle Income
58	IRQ	Upper Middle Income	119	ZAF	Upper Middle Income
59	ISL	High Income	120	ZMB	Low Income
60	ISR	High Income	121	ZWE	Lower Middle Income
61	ITA	High Income			

Table S5. Variable selection and data sources.

No.	Variable	Indicator	Selection reasons	Data sources
1	Economy	GDP (constant 2015 US$)	Countries capable of large-scale production often export goods to smaller countries that can't produce the same goods as cost-effectively, leveraging the economies of scale to produce goods more cheaply.	World Bank
2	Population	Total population	The size and characteristics of a country's population can affect the demand for certain goods, influencing trade flows.	World Bank
3	Government effectiveness	Government Effectiveness: Percentile Rank, Upper Bound of 90% Confidence Interval	Efficient governance often leads to more streamlined trade regulations and procedures, making it easier for businesses to export and import goods. Efficient governance can boost investor confidence, attracting foreign direct investment that can stimulate trade.	World Bank
4	Access to Internet	Proportion of population covered by at least a 2G mobile network	The internet, by facilitating e-commerce, expands the potential for international trade, enabling businesses to reach foreign customers with ease and facilitating faster, more efficient communication between businesses, customers, and suppliers worldwide.	World Bank
5	Performance of export sectors	Export value index (2000 = 100)	The Export Value Index serves as a measure of a country's export sector performance. An upward trend in this index signifies growth in the value of a country's exports, while a downward trend indicates a decline in export value.	World Bank
6	Technology level	Scientific and technical journal articles	Advanced technology can boost productivity and efficiency, reduce production costs, and enable countries to export more goods and services. It can also foster innovation, creating new products and industries that generate new trade flows.	World Bank

Table S6. Explanatory variables and selection rationale for 55 SDG indicators. The first column, labeled 'SDG No.,' corresponds to the SDG indicator listed in Table A.1. As an illustration, SDG No.1 pertains to the indicator 'Proportion of Population Using Basic Drinking Water Services, by Location (%).' The explanatory variables for this particular indicator encompass factors such as economic development, environmental protection, education, governance, and population density. The following indicators represent distinct aspects of a nation's development: "GDP per capita" for economic development, "comparative advantage in environmental goods" for environmental protection, "employment to population ratio" for social development, "proportion of total government spending on education" for education, "scientific and technical journal articles" for technology, "domestic general government health expenditure per capita" for healthcare, "government effectiveness (percentile rank)" for governance, "population density" for population, "foreign direct investment, net inflows (% of GDP)" for openness, "value added of agriculture, forestry, and fishing per agricultural land" for agricultural productivity, and "proportion of population covered by at least a 2G mobile network" for access to the internet.

SDG No.	Economic development	Environmental protection	Social development	Education	Technology	Healthcare	Governance	Population density	Openness	Agriculture productivity	Access to the Internet
1	Economic development level can improve water services by providing the financial resources necessary to invest in infrastructure, technology, and human resources needed to ensure access to clean and safe water.	Protection and sustainable management of water resources, including watersheds, aquifers, and ecosystems, are essential for maintaining the quality and availability of drinking water.	-	Improving public awareness about the importance of water conservation and hygiene can help reduce water wastage and contamination, thereby improving access to safe drinking water	-	-	Effective policies and regulations of government can promote the efficient use of water resources, improve water quality, and ensure equitable access to safe drinking water.	High population density can put pressure on water resources and infrastructure, making it more difficult to provide basic drinking water services to all.	-	-	-
2	Economic development level can play a key role in	Protection and sustainable management of water resources,	-	Improving public awareness about the	-	-	Efficient governments can allocate	High population density can put pressure	-	-	-

	improving water services by providing the financial resources necessary to invest in infrastructure, technology, and human resources needed to ensure access to sanitation services.	including watersheds, aquifers, and ecosystems, are essential for maintaining the quality and availability of drinking water.		importance of water conservation and hygiene can help reduce water wastage and contaminati on, thereby improving access to safe drinking water			resources wisely, ensuring the availability of funds to improve sanitation facilities, education, and public awareness campaigns.	on water resources and infrastructur e, making it more difficult to provide sanitation services to all.			
3	Economic development enables governments to invest in public health interventions, such as nutrition education and iron supplementation programs, and supports the development of infrastructure that contributes to better health outcomes.	Environmental factors, such as pollution and contamination of water and food sources, can contribute to anemia by affecting nutrient availability and absorption.	-	-	-	Effective health policies and programs that focus on improving nutrition, providing iron supplement ation, and addressing the risk factors of anemia can help reduce its prevalence among women.	-	-	-	Increased agricultur al productiv ity often leads to more food availabilit y and diversity. This can improve the nutritiona l intake of women, including iron, which is critical for preventin g	-

										anaemia.	
4	Economic development enables governments to invest in public health interventions, such as nutrition education and iron supplementation programs, and supports the development of infrastructure that contributes to better health outcomes.	Environmental factors, such as pollution and contamination of water and food sources, can contribute to anemia by affecting nutrient availability and absorption.	-	-	-	Effective health policies and programs that focus on improving nutrition, providing iron supplementation, and addressing the risk factors of anemia can help reduce its prevalence among women.	-	-	-	Increased agricultural productivity often leads to more food availability and diversity. This can improve the nutritional intake of women, including iron, which is critical for preventing anaemia.	-
5	Economic development often comes with increased public and private investment in infrastructure. This can include irrigation systems, post-	-	-	Education equips farmers with the necessary knowledge and skills to implement more efficient farming	Advances in biotechnology can lead to the development of high-yield and disease-resistant crop varieties.	-	-	High population density could decrease cereal yield per hectare if the pressure on land resources	Countries that are more open might have greater access to high-quality seeds, machinery, and farming techniques that can	Higher agricultural productivity could result from better farming techniques,	Internet access can play a significant role in cereal yield because it can facilitate access to informatio

	harvest storage, and transportation systems that can reduce losses and enhance the efficiency of the agricultural sector.			practices.	These improved seeds can lead to significantly higher cereal yields.			leads to over-farming or farming on marginal lands that aren't as fertile. However, high population density could also potentially increase cereal yield by adopting intensive farming techniques.	increase yield.	improved seeds, and more effective use of inputs like fertilizer and irrigation, which would likely increase cereal yield.	n on better farming practices, weather forecasts, and market prices.
6	Poverty can influence maternal health and contribute to higher maternal mortality ratios. Improving overall living conditions can help reduce maternal mortality.	-	-	Women's education and awareness about reproductive health, family planning, and safe childbirth practices play a significant role in reducing maternal mortality.	Technological innovations like telemedicine, mobile health applications, and advanced diagnostic tools can facilitate early detection of complications, provide critical health	Limited access to healthcare facilities, skilled healthcare professionals, and essential medical supplies can contribute to higher maternal mortality ratios.	-	-	-	-	-

					information, and improve patient care.						
7	A higher level of economic development can result in better healthcare infrastructure, more educated population, improved living conditions, and increased access to nutritious food and clean water.	-	-	Educated mothers are more likely to understand and seek out healthcare services, follow recommended health practices, and provide better care for their infants.	-	Access to quality prenatal and postnatal care, immunizations, well-child care, and emergency medical services can significantly reduce the chances of infant death.	Effective government implementation and enforcement of policies related to healthcare, education, economic development, and social services is crucial for reducing infant mortality, as inefficiencies or corruption can hinder these services, leading to higher infant mortality rates.	-	-	-	-
8	Economic development can boost healthcare infrastructure, education access, living conditions, and	Environmental factors, like high levels of air and water pollution or lack of access to clean water and sanitation, can affect neonatal	-	Educated mothers, more likely to prioritize prenatal care, adhere to health guidelines	-	Access to quality prenatal and postnatal healthcare, ensuring maternal	Government effectiveness in formulating and implementing policies tied to	-	-	-	-

	nutritional standards, collectively improving maternal and neonatal wellbeing and thus reducing neonatal mortality.	health and contribute to neonatal mortality.		during pregnancy, and identify potential health issues, along with having better health behaviors and resource access, can positively influence neonatal health.		health during pregnancy and providing immediate newborn care, early complication detection, and prompt medical intervention, is crucial for reducing neonatal mortality.	maternal and neonatal health, healthcare accessibility, education, economic development, and social welfare significantly influences the neonatal mortality rate.				
9	Economic development, impacting general living conditions like housing and nutrition, can influence tuberculosis incidence.	-	-	Educated individuals are more likely to seek medical help for persistent coughs or other tuberculosis symptoms, adhere to treatment protocols, and understand how to reduce the risk of transmission	-	The availability and quality of healthcare services play a crucial role in controlling the incidence of tuberculosis.	Effective government policies and programs, encompassing health initiatives and social protection measures addressing tuberculosis's social determinants, are essential for tuberculosis control.	In densely populated areas, tuberculosis can spread more easily because the bacterium that causes tuberculosis is transmitted from person to person through the air.	-	-	-

				to others.							
10	Countries with a higher level of economic development may have the resources to invest in longer periods of primary education.	-	Social development, including societal attitudes towards education, can influence the duration of primary education. In societies where education is highly valued, children are more likely to complete the full duration of primary education. Moreover, societal structures and attitudes towards gender can also play a part. For example, in some societies, girls might be encouraged to leave school early for	Societal education levels, especially adult literacy and parental education, influence primary education duration, as educated parents, understanding education's value, are likelier to encourage prolonged schooling for their children.	-	-		-	-	-	-

			marriage or other domestic responsibilities.								
11	Economic development, linked to higher preprimary school enrollment, typically allows wealthier countries and higher-income families to afford early childhood education, especially when not fully government-subsidized.	-	Factors such as the societal recognition of the importance of early education, gender equality, and family support systems can influence enrollment rates.	Parental education levels in a society can influence preprimary school enrollment, as more educated parents, understanding early childhood education's value, are likelier to enroll their children in preprimary education.	-	Access to quality healthcare, ensuring children's health for school attendance and providing platforms to promote and facilitate school enrollment, can both directly and indirectly influence preprimary school enrollment.	Efficient governments can impact preprimary school enrollment by investing in early education, enforcing laws, ensuring quality, and providing support through public or subsidized private education, thereby facilitating increased school attendance.	Population density influences preprimary school enrollment; dense areas may have more resources, easing enrollment, but can strain resources, decreasing education quality, while sparse areas may face distance-related enrollment barriers.	-	-	Internet access can boost preprimary school enrollment, particularly in regions with scarce traditional education resources, by providing parents with crucial information about the importance of early education, available schools, and enrollment procedures.
12	Economic development can increase access to education, reducing poverty, and	-	Factors such as the societal recognition of the importance of early education,	The overall education level in a society, especially among parents,	-	-		In densely populated areas, the availability of more schools can potentially	-	-	The internet can provide information about the importance of

	promoting gender equality.		gender equality, and family support systems can influence enrollment rates.	influences preprimary school enrollment as more educated parents, understanding the value of early childhood education, are likely to enroll their children in preprimary education.				lead to a higher GPI by facilitating school attendance for both genders, though overcrowding and the differential impact on boys and girls could affect this index.			education for all children, regardless of gender.
13	Economically developed countries often have more resources to implement and enforce equitable laws and regulations.	-	Social development influences societal attitudes towards gender roles, affecting laws and regulations for women's economic inclusion, with more developed societies typically being more open to gender equality.	A society's higher education level can foster understanding of gender equality, influencing public opinion to pressure governments towards equitable laws and regulations.	-	-	Government effectiveness in enforcing laws, commitment to gender equality, and resistance to corruption shape the legal environment for women's economic inclusion.	-	-	-	Internet access facilitates awareness and advocacy of gender equality issues, enables economic opportunities for women like online work, and empowers them to advocate for legal reforms.

14	In less economically developed countries, the prevalence of small-scale family businesses and farms often necessitates family contributions, whereas economic development shifts employment towards formal, wage-paying jobs, decreasing the proportion of women's employment constituted by family work.	-	Traditional gender norms may compel women to work unpaid in family businesses or farms, an issue that can be mitigated by societal progression towards gender equality.	Education elevates women's likelihood of formal employment over unpaid family work and bolsters their advocacy for fair compensation.	-	-	Government policies promoting gender equality, women's education, and formal employment opportunities can significantly affect the percentage of women's work that is unpaid family contributions.	-	Greater openness fosters economic opportunities and job creation, potentially reducing women's reliance on family work by providing formal employment options and facilitating knowledge and technology transfer that can boost productivity and introduce new jobs.	In agriculture-dominant, low-productivity countries, women's family farming contributions are common, but agricultural improvements could facilitate their shift to formal employment.	Internet access can provide women with job resources and opportunities for remote work, e-commerce, and online entrepreneurship
15	-	-	Societal attitudes towards gender equality dictate the proportion of parliamentary seats held by women, with progressive societies encouraging	Education empowers women for political participation by increasing political awareness and self-confidence, while societies	-	-	Government effectiveness in promoting gender equality, through policies like gender quotas for parliamentary seats and ensuring	-	-	-	-

			political participation and patriarchal ones posing more barriers.	with higher education levels tend to be more accepting of women in politics.			free and fair elections, greatly influences women's political representation.				
16	Economic development, crucial for sanitation infrastructure, can lead to lower open defecation rates in wealthier countries.	-	Social development can alter societal attitudes towards sanitation, with education and awareness challenging norms where open defecation is socially acceptable, thus aiding its elimination.	Higher education levels increase awareness of the health risks of open defecation and benefits of sanitation, empowering people to demand better governmental services, reducing open defecation.	Technological advancements provide affordable sanitation alternatives to open defecation, particularly where traditional systems aren't feasible, facilitating better facilities and reducing open defecation.	-	Government effectiveness in providing sanitation infrastructure, maintaining systems, and conducting education campaigns significantly influences open defecation rates and social norms surrounding it.	Population density affects open defecation rates, with densely populated urban areas prioritizing sanitation due to immediate health impacts, while less dense rural areas may deprioritize it.	-	-	Internet access can reduce open defecation by spreading awareness about sanitation importance and dangers, and facilitating information dissemination about sanitation programs.
17	Economic development can enhance water-use efficiency, as developed economies can invest in efficient	-	-	Higher levels of education can lead to a better understanding of the importance of water	Advanced technologies can enable more precise water usage, minimizing waste.	-	Government effectiveness in managing water resources, including implementing water-	-	-	Agriculture's water usage significantly impacts water efficiency; more	-

	technologies and potentially shift from water-intensive industries like agriculture to less water-intensive sectors.			conservation and efficiency. Educated individuals may be more likely to implement water-saving practices, both at home and in businesses.			saving policies, investing in infrastructure, and enforcing regulations, greatly influences water use efficiency.			productive practices or crops and efficient irrigation techniques can increase output per water unit and reduce waste.	
18	Developed countries may have high-consuming industries, and they may invest in water-saving technologies and infrastructure to mitigate stress.	Environmental protection, including safeguarding watersheds and wetlands integral to the water cycle, ensures freshwater resources' sustainability, thus reducing water stress.	-	-	-	-	Government effectiveness in managing water resources, including implementing conservation laws, investing in infrastructure, and managing demand, greatly influences water stress levels.	High population density increases freshwater demand for domestic and industrial use, potentially escalating water stress, particularly if available freshwater resources are limited.	-	Agriculture, being a major freshwater consumer, impacts water stress significantly; efficient practices and irrigation methods can reduce freshwater withdrawal, thus lessening water	-

										stress.	
19	Economic development can strain water resources, potentially reducing areas with permanent water bodies, while also enabling investment in environmental protection and sustainable water management, helping to preserve these water bodies.	Environmental protection efforts help preserve existing water bodies and prevent habitat loss due to threats like pollution and climate change, maintaining the health and size of permanent bodies of water.	-	-	-	-	Government effectiveness in managing natural resources and enforcing environmental protection measures can influence the land area covered by permanent bodies of water through policies on water management, land use, and pollution control.	High population density can influence urbanization and land use, potentially affecting the total area of water bodies due to construction or creation of new water bodies for urban design or flood management.	-	Agricultural productivity, reliant on extensive irrigation, can alter water bodies; overuse can deplete lakes and rivers, while effective water management can help maintain them.	-
20	Economic development can impact seasonal water-filled areas either negatively through industrial activities and urban	Environmental protection measures preserve seasonal water bodies, crucial for habitats, the local water cycle, and soil health, whereas practices like deforestation or	-	-	-	-	Government capability to enforce environmental and land use regulations significantly influences the land	High population density can influence urbanization and land use, potentially affecting the total area of	-	Agricultural productivity, reliant on extensive irrigation, can alter water bodies;	-

	development, or positively by providing resources for better environmental protection and sustainable practices.	pollution disrupt them; strong policies help maintain these areas.					area covered by seasonal water bodies, with policies preventing overdevelopment or degradation of these areas.	water bodies due to construction or creation of new water bodies for urban design or flood management.		overuse can deplete lakes and rivers, while effective water management can help maintain them.	
21	Economic development strongly predicts electricity access; as countries develop, they typically build necessary infrastructure, including power systems, and households can afford the associated costs.	-	-	-	Technology level impacts electricity access; advanced technology can cost-effectively extend access to remote areas, with renewable energy technologies like solar panels providing electricity where traditional grid extension isn't feasible.	-	Government effectiveness is crucial in electricity provision, affecting infrastructure, sector regulation, and access policies. Ineffectiveness may lead to infrastructure inadequacy and uneven access.	Population density affects the cost-effectiveness of grid extension; urban areas with high density typically have lower per-household electricity provision costs than rural areas, often leading to rural electrification lag.	-	-	-

22	Wealthier households and countries are more likely to afford and adopt cleaner but often pricier fuels and technologies.	-	-	-	The availability and advancement of clean technologies can influence their adoption; a higher technology level can lead to more efficient, cost-effective clean technologies.	-	Government policies are crucial in encouraging clean fuels and technology use, including clean energy subsidies, air quality regulations, or rural electrification programs.	-	-	-	Internet access facilitates the information spread about clean fuels and technologies' benefits and use, and enables online purchasing of clean technologies not available locally.
23	As countries develop, they typically invest more in renewable technologies and infrastructure, and wealthier economies with higher energy demands often shift towards sustainable sources due to environmental concerns.	Environmental protection efforts can increase renewable energy share through urgent push for sustainable sources, mitigating climate change, protecting air quality, and conserving resources.	-	-	The technology level can impact renewable energy share; advanced technologies enhance efficiency and cost-effectiveness of renewable sources, and improve harnessing, storage, and	-	Government policies, regulations, and effectiveness in implementation play a critical role in renewable energy adoption, influencing mandates, subsidies, carbon pricing, and grid access	-	-	-	-

					distribution.		for renewable sources.				
24	Economic development can influence energy intensity by shifting from energy-intensive industries to less intensive sectors and investing in energy-efficient technologies and practices.	-	-	Education influences energy intensity by increasing energy-efficiency awareness, enhancing the ability to use efficient technologies, and fostering innovation in energy-efficient technologies.	Technology level significantly affects energy intensity; advanced technologies enable more efficient energy use in industries, buildings, and transportation, including high-efficiency equipment, energy-saving appliances and vehicles.	-	Government policies and their effective implementation can influence energy intensity through efficiency standards for buildings, appliances, vehicles, and industry practices.	-	-	-	-
25	Economic development influences the number of bank branches; as an economy grows, corresponding banking sector expansion	-	Higher social development often correlates with a more mature banking sector; as societies	Higher education levels can create greater demand for banking services, as more	Online and mobile banking services might reduce the need for physical branches,	-		Population density impacts the location and number of bank branches; more branches	Countries open to international trade and investment may have a more developed banking	-	The rise of online and mobile banking allows customers to perform tasks without

	supports increased economic activity and wealthier populations demand more banking services.		develop, increased demand for financial services usually leads to a higher number of bank branches.	educated individuals may have higher incomes, savings, and investment needs, and a better understanding of banking services.	but technology can also enhance banking operations' efficiency and cost-effectiveness, potentially enabling more branches in new areas.			may open in densely populated areas due to a larger customer base.	sector due to increased economic activity and foreign banks' presence, but openness could also lead to banking sector consolidation and fewer, larger branches due to international competition.		visiting a branch, potentially reducing physical branches' need, but internet access can increase awareness and comfort with banking services, potentially boosting overall demand.
26	Economic development can shift from material-intensive industries like manufacturing to less intensive sectors like services, reducing material consumption per GDP unit.	Environmental protection efforts can decrease material intensity by pushing for more efficient resource use, involving resource conservation, biodiversity protection, or climate change mitigation.	-	-	Technology level significantly affects material intensity; advanced technologies enable more efficient material use in production across various sectors, including high-	-	Government policies can influence material intensity, including regulations and incentives promoting material efficiency, waste reduction, recycling, and product design standards.	-	Openness affects the availability and price of materials; more open countries might import more materials, increasing domestic consumption, while increased exports of raw materials due to openness	-	Internet access can inform consumers about sustainable consumption, potentially reducing material use, while online shopping impacts material consumption through product

					efficiency industrial equipment and recycling and waste management technologies.				could potentially reduce domestic consumption.		choice, packaging, and delivery.
27	As economies develop and incomes rise, individuals consume more goods, but higher incomes also enable more efficient and sustainable consumption patterns.	Environmental protection efforts can affect material consumption per capita; environmental impact awareness can lead to sustainable consumption patterns, like choosing products with less packaging or made from recycled materials.	-	-	A country's technology level can affect its material intensity; advanced technologies enable efficient material use, reducing consumption per GDP unit, while outdated technologies may result in wasteful use.	-	Government policies influence material consumption per capita, including those promoting material efficiency, waste reduction, and recycling, and regulations affecting material price and availability.	Urban dwellers in densely populated areas may consume fewer goods due to smaller living spaces, but urban lifestyles can also lead to high consumption of certain materials like construction materials.	More open countries might import more materials, increasing domestic consumption, while increased raw material exports due to openness could potentially reduce domestic consumption.	-	The internet influences consumer behavior, providing information about sustainable consumption, potentially reducing material use if consumers opt for sustainable products. Online shopping impacts material consumption through product choice, packaging, and

											delivery specifics.
28	As economies develop and industrialize, there's a shift from self-employment towards wage and salaried work in larger businesses and industries.	-	-	Education level can affect this indicator as well. More educated populations may have more opportunities for wage and salaried work, particularly in skilled professions.	-	-	Government policies and regulations can significantly impact the structure of the labor market. This can include labor laws, minimum wage policies, and regulations related to job security and working conditions. The effectiveness of a government in implementing and enforcing these policies can influence the proportion of wage and salaried workers.	High population density areas, often associated with urbanization, tend to have a higher proportion of wage and salaried workers compared to rural areas where self-employment and informal employment are common, due to differing prevalent industries.	-	-	The internet can facilitate finding wage and salaried jobs through online job postings or professional networking sites, potentially increasing the proportion of wage and salaried workers.
29	As economies develop, there is	-	-	Education influences	A country's technology	-	Government policies	-	Openness to foreign direct	-	-

	often a shift from agriculture to manufacturing (and later to services), according to the typical pattern of structural transformation.			the manufacturing sector by providing skills needed for various jobs.	level can impact its manufacturing sector's size and productivity; advanced technologies enable efficient production, leading to higher value added, including high-tech equipment and supply chain software.		significantly influence the manufacturing sector, including trade, taxes, infrastructure, education, and innovation policies; effective policy implementation and enforcement can significantly affect manufacturing value added.		investment influences the manufacturing sector by bringing capital, technology, and expertise that boost the sector and stimulate competition and innovation.		
30	As economies develop, shifts from labor-intensive sectors to capital-intensive ones may decrease labor's GDP share, but the effect depends on factors like wage levels, worker bargaining power, and economic	-	-	Higher education levels can increase workers' bargaining power and potentially wages, but if employers mainly capture the benefits of higher skills, the labor share	Advanced technologies can increase productivity and potentially wages, but if technology substitutes labor and the benefits of technological progress	-	Government policies can influence the labor share of GDP, including labor laws, minimum wage policies, tax policies, and job security and working conditions regulation	Urbanization, often linked to higher population densities, might increase the labor share of GDP by leading to more industrial and service jobs with higher	Countries more open to trade and foreign investment might see an increase in the labor share of GDP due to potential increases in high-wage jobs from foreign companies	-	The internet can increase labour productivity by enabling more efficient ways of working, which could potentially increase wages and

	structure.			might not increase.	are mainly captured by capital owners, the labor share could decrease.			wages, but an oversupply of labor could decrease the labor share if not matched by job growth.	paying higher wages than domestic ones.		the labour share of GDP.
31	Economic conditions can significantly influence the number of refugees from a country. High levels of poverty, unemployment, or lack of opportunity can push people to leave their country in search of better economic prospects.	-	Levels of social development, including factors such as social cohesion, equality, and justice, can influence the number of refugees. Societies with high levels of conflict, discrimination, or social inequality may produce more refugees.	-	-	-	Government effectiveness can greatly influence the number of refugees from a country. Factors such as political instability, conflict, poor governance, or human rights abuses can lead to large numbers of people fleeing a country.	-	-	-	-
32	Economic development often leads to urbanization as people seek better job opportunities,	-	-	-	Advancements in transportation and communication technologie	-	Government policies can influence urbanization rate and pattern, including	-	-	High agricultural productivity can slow urbanizati	-

	higher wages, and improved living conditions, typically resulting in a shift from agriculture to urban-based manufacturing and services.				s can facilitate urban growth, while technologies improving agricultural productivity or enabling remote work might slow urbanization.		land use, housing, transportation, and infrastructure policies.			on by making rural life more viable, while low productivity might lead to rural-urban migration as people seek better opportunities.	
33	Economic development often leads to urbanization and increased urban population growth rate as a shift occurs from agriculture to city-centered manufacturing and services for better job opportunities and living conditions.	-	-	-	Transportation technology improvements can boost migration to urban areas, while technologies enhancing agricultural productivity or enabling remote work may slow urban population growth by improving rural economic	-	Government policies, including those related to land use, housing, transportation, and infrastructure, can influence the urban population growth rate	Population density can affect urban population growth, with densely populated areas potentially facing constraints like limited land availability, while areas with low population density may have more room for urban	-	High productivity can enhance rural economic viability, potentially slowing rural-urban migration, while low productivity might increase migration as people seek better opportuni	-

					viability.			expansion.		ties.	
34	Industrialization and urbanization, often linked to economic development, can increase air pollution, but further economic development can also provide resources for pollution control and cleaner production technologies.	Environmental protection efforts can greatly affect levels of fine particulate matter, including measures to reduce emissions from factories, power plants, and vehicles, and efforts to promote cleaner energy sources.	-	Higher education levels can increase awareness about health risks associated with fine particulate matter and benefits of pollution reduction measures, empowering people to demand better environmental regulations.	Advanced technologies can enable cleaner production processes and more efficient energy use, reducing emissions.	-	Government policies and regulations can impact fine particulate matter levels, including air quality standards, emissions regulations, and enforcement efforts.	Population density can influence fine particulate matter levels; in densely populated urban areas, emissions from vehicles, heating, and other sources can be concentrated, leading to higher particulate matter levels.	Countries more open to international trade and investment might reduce particulate matter levels by adopting stricter environmental standards to align with international norms, but unmanaged increase in economic activity due to openness can also raise pollution.	-	The internet can spread awareness about the dangers of air pollution and ways to mitigate it, potentially leading to behaviors or policies that reduce particulate matter levels.
35	Industrialization and urbanization, often linked to economic development, can increase air pollution, but further economic development can also provide resources for pollution control	Environmental protection efforts can greatly affect levels of fine particulate matter, including measures to reduce emissions from factories, power plants, and vehicles, and efforts to promote cleaner energy sources.	-	Higher education levels can increase awareness about health risks associated with fine particulate matter and benefits of pollution reduction	Advanced technologies can enable cleaner production processes and more efficient energy use, reducing emissions.	-	Government policies and regulations can impact fine particulate matter levels, including air quality standards, emissions regulations, and	Population density can influence fine particulate matter levels; in densely populated urban areas, emissions from vehicles, heating, and	Countries more open to international trade and investment might reduce particulate matter levels by adopting stricter environmental standards to align with international	-	The internet can spread awareness about the dangers of air pollution and ways to mitigate it, potentially leading to behaviors

	and cleaner production technologies.			measures, empowering people to demand better environment al regulations.			enforcement efforts.	other sources can be concentrate d, leading to higher particulate matter levels.	norms, but unmanaged increase in economic activity due to openness can also raise pollution.		or policies that reduce particulate matter levels.
36	Industrialization and urbanization, often linked to economic development, can increase air pollution, but further economic development can also provide resources for pollution control and cleaner production technologies.	Environmental protection efforts can greatly affect levels of fine particulate matter, including measures to reduce emissions from factories, power plants, and vehicles, and efforts to promote cleaner energy sources.	-	Higher education levels can increase awareness about health risks associated with fine particulate matter and benefits of pollution reduction measures, empowering people to demand better environment al regulations.	Advanced technologie s can enable cleaner production processes and more efficient energy use, reducing emissions.	-	Government policies and regulations can impact fine particulate matter levels, including air quality standards, emissions regulations, and enforcement efforts.	Population density can influence fine particulate matter levels; in densely populated urban areas, emissions from vehicles, heating, and other sources can be concentrate d, leading to higher particulate matter levels.	Countries more open to international trade and investment might reduce particulate matter levels by adopting stricter environment al standards to align with international norms, but unmanaged increase in economic activity due to openness can also raise pollution.	-	The internet can spread awareness about the dangers of air pollution and ways to mitigate it, potentially leading to behaviors or policies that reduce particulate matter levels.
37	Many developing economies heavily depend on exporting natural resources,	Environmental protection policies can influence the extraction and use of natural resources, and thus natural resources	-	Higher education levels can lead to a more diversified economy,	Technologic al advancemen ts can increase resource extraction	-	Government policies and effectivenes s in managing natural resources	High population density can increase natural resource demand and	Openness to foreign direct investment, particularly in developing countries,	-	-

	resulting in a high GDP percentage from resource rents, which may decrease as economies diversify and develop.	rents. Strict environmental regulations might limit the exploitation of natural resources, reducing their contribution to GDP. Conversely, lack of environmental protection could result in over-exploitation of natural resources, potentially leading to a temporary increase in natural resources rents as a percentage of GDP.		reducing dependence on natural resource rents, and educated populations might demand better natural resource management and use, potentially influencing natural resource rents.	efficiency, potentially increasing natural resource rents, but can also foster development and adoption of alternative, non-resource-based industries, potentially reducing reliance on natural resource rents.		can influence natural resource rents, including policies related to resource extraction, taxation, and export, as well as efforts to prevent illegal resource exploitation.	rents, but it can also drive economic diversification and reduced resource reliance, influenced by urbanization and industrial development.	can increase natural resource extraction and potentially their GDP contribution.		
38	Economic development can shift focus from material-intensive industries to less material-intensive sectors, reducing material consumption per GDP unit, and enable investment in efficient technologies and	Environmental protection efforts can decrease material intensity by pushing for more efficient resource use, including resource conservation, biodiversity protection, or climate change mitigation.	-	-	Advanced technologies enable more efficient material use in production processes across various economic sectors, from high-efficiency industrial equipment	-	Government policies, including regulations and incentives for material efficiency, waste reduction and recycling, can significantly affect material intensity,	-	Openness can affect the availability and price of materials; more open countries might import more materials, increasing domestic material consumption, whereas increased exports of	-	The internet, by providing information about sustainable consumption and environmental impact, and influencing product choice and packaging through online

	practices.				to recycling and waste management technologies.		with effectiveness in policy implementation and enforcement playing a crucial role.		raw materials due to openness could potentially reduce domestic consumption.		shopping, can potentially increase or decrease material consumption depending on consumer choices and delivery specifics.
39	Economic development can shift focus from material-intensive industries to less material-intensive sectors, reducing material consumption per capita, and enable investment in efficient technologies and practices.	Environmental protection efforts can decrease material intensity by pushing for more efficient resource use, including resource conservation, biodiversity protection, or climate change mitigation.	-	-	Advanced technologies enable more efficient material use in production processes across various economic sectors, from high-efficiency industrial equipment to recycling and waste management technologies.	-	Government policies, including regulations and incentives for material efficiency, waste reduction and recycling, can significantly affect material intensity, with effectiveness in policy implementation and enforcement playing a crucial role.	Population density can impact per capita material consumption, with densely populated urban areas potentially consuming fewer material goods, but urban lifestyles can also increase consumption of specific materials like construction materials.	Openness can affect the availability and price of materials; more open countries might import more materials, increasing domestic material consumption, whereas increased exports of raw materials due to openness could potentially reduce domestic consumption.	-	The internet, by providing information about sustainable consumption and environmental impact, and influencing product choice and packaging through online shopping, can potentially increase or decrease material consumption

											depending on consumer choices and delivery specifics.
40	Economic development can influence the number of people affected by floods by providing resources for effective flood risk management, but it can also increase flood risks through environmental changes from economic activities like deforestation and land degradation.	Environmental protection efforts can influence the number of people affected by floods. For instance, the preservation of natural flood barriers like wetlands and forests can reduce flood risks. On the other hand, environmental degradation, such as deforestation and soil erosion, can increase flood risks.	Social development can also enhance community resilience and the effectiveness of disaster response, potentially reducing the number of people affected by floods.	Effective healthcare services can mitigate the impact of floods by providing timely medical aid to affected individuals, potentially reducing the number of people seriously affected.	The level of technology can also affect the number of people affected by floods. Advanced technologies can aid in flood forecasting, early warning, and emergency response, potentially reducing the number of people affected by floods. Furthermore, engineering technologies can be used to construct flood barriers and	-	Government policy effectiveness can significantly impact the number of people affected by floods, including land use, urban planning, disaster risk reduction, and climate change adaptation policies, and effective early warning systems and emergency response capacities.	Population density, especially in flood-prone areas, can influence the number of people affected by floods; a single flood event in densely populated areas can affect many people, and unmanaged urbanization can increase flood risks.	-	-	The internet can facilitate early warning systems and rapid communication during flood events, helping people to evacuate or take other protective measures.

					design flood-resistant infrastructure.						
41	As economies grow and industrialize, CO2 emissions also rise. However, as economies become more efficient and potentially transition to cleaner sources of energy, emissions may stabilize or decrease.	Policies and practices that promote the sustainable use of resources, reduce waste, and encourage the adoption of renewable energy can lead to lower CO2 emissions per capita.	-	-	Advanced technologies can enable more efficient energy use and the adoption of renewable energy sources, reducing CO2 emissions. Conversely, reliance on outdated, inefficient technologies can lead to higher emissions.	-	Government policy effectiveness can impact the number of people affected by floods, including land use, urban planning, disaster risk reduction, and climate change adaptation policies.	-	-	-	-
42	Economic growth can increase fish and seafood demand, potentially increasing capture fisheries production, but can also provide resources for sustainable	Overfishing, pollution, and habitat destruction can reduce fish stocks and capture fisheries production growth rate, whereas effective conservation and management measures can help maintain or	-	Higher education levels could lead to better knowledge about sustainable fishing practices, potentially leading to	Advanced technologies can enable more efficient and sustainable fishing practices. However, if not managed properly,	-		High population density areas might have higher fish demand, potentially increasing fishing efforts and capture	Open access to international markets can increase fish and seafood demand, potentially increasing capture fisheries production,	High agricultural productivity could potentially reduce the pressure on fishing if people	-

	fishing practices and alternative livelihoods, potentially reducing pressure on fish stocks.	increase fish stocks.		more stable or even increased growth rates.	these technologies can also lead to overfishing.			fisheries production growth rates, but overfishing due to high demand could also deplete fish stocks and reduce long-term growth rate.	but this must be balanced with sustainable fishing practices to prevent overfishing.	can obtain sufficient food from agriculture, potentially leading to more sustainable growth rates of capture fisheries production.	
43	Growing economies may increase fish and seafood demand, leading to increased aquaculture production, and economic development can provide the infrastructure and resources necessary for aquaculture expansion and modernization.	Sustainable practices can prevent environmental degradation and ensure industry's long-term viability, while poor environmental practices can lead to disease outbreaks, biodiversity loss, and other issues limiting aquaculture growth.	-	Higher levels of education could lead to better understanding of sustainable and efficient aquaculture practices, potentially increasing the growth rate of aquaculture production.	Innovations in breeding, disease control, feed efficiency, and waste management can all increase the productivity and sustainability of aquaculture, leading to higher growth rates.	-	Government policies and regulations, including those related to licensing, environmental regulations, disease management, and technological advancement support, can affect the growth rate of aquaculture production, with the	High population density could increase fish and seafood demand, potentially driving up aquaculture production growth, but space constraints or environmental challenges like pollution in densely populated	-	High agricultural productivity might free resources like land and water for aquaculture, potentially increasing its growth rate, but if it leads to environmental	-

							effectiveness of implementation and enforcement playing a significant role.	areas might limit aquaculture growth.		issues like water pollution from agricultural runoff, this could negatively impact aquaculture production.	
44	Economic growth may increase fish and seafood demand, leading to increased fisheries production, and economic development can provide the infrastructure and resources necessary for both capture fisheries and aquaculture expansion and modernization.	Overfishing, pollution, and habitat destruction can reduce fish stocks and capture fisheries growth, but effective conservation and sustainable aquaculture practices can maintain fish stocks and ensure long-term industry viability.	-	Higher education levels could lead to better understanding of sustainable fishing and aquaculture practices, potentially increasing the growth rate of total fisheries production.	Advanced technologies can enable more efficient and sustainable fishing practices and improve aquaculture productivity, potentially leading to higher growth rates.	-	Government policies and regulations, including those for both capture fisheries and aquaculture, can significantly impact total fisheries production growth, with the effectiveness of policy implementation and enforcement being crucial.	High population density could boost fish and seafood demand, potentially driving total fisheries production growth, but challenges like overfishing or space constraints for aquaculture in densely populated areas could limit growth.	Trade policies can influence total fisheries production growth; international market access can boost fish demand, but must be balanced with sustainable practices to ensure long-term industry sustainability.	High agricultural productivity could free resources for aquaculture, potentially boosting fisheries production, but related environmental issues like water pollution could negatively impact both wild	-

										fisheries and aquaculture.	
45	Economic activities like agriculture, logging, and urbanization can cause deforestation, but as economies develop, they may also invest more in forest conservation, sustainable land use, and reforestation initiatives.	Conservation initiatives, reforestation programs, and measures to prevent deforestation can increase forest area. Conversely, lack of environmental protection can lead to deforestation and reduction in forest area.	-	-	-	-	Government policies and regulations, including land use, forest conservation, and reforestation policies, and their enforcement can significantly impact the forest area, with government effectiveness in policy implementation and enforcement playing a crucial role.	Areas with high population density can experience more pressure to clear forests for urban development or agriculture, while areas with low population density might have less pressure on forests, potentially leading to a higher forested land percentage.	-	Agricultural productivity, particularly in agriculture-dominant economies, can influence forest area, with high productivity reducing deforestation for land expansion, and low productivity potentially increasing it.	-
46	Economic growth can fund the establishment and maintenance	Environmental protection efforts can impact the proportion of Key Biodiversity Areas	-	-	-	-	The effectiveness of government agencies in	-	-	-	-

	of protected areas but can also drive activities like deforestation, infrastructure development, and resource extraction that might encroach on Key Biodiversity Areas.	covered by protected areas, balancing the risks of encroachment from economic growth-driven activities.					designating, managing, and enforcing protected areas can significantly affect the proportion of Key Biodiversity Areas they cover.				
47	Economic development can impact mountain biodiversity both positively, by creating jobs and improving living standards, and negatively, through unsustainable practices that destroy habitats and disrupt ecosystems.	Environmental protection, crucial for preserving fragile mountain biodiversity, involves protecting Mountain Key Biodiversity Areas from human activities and establishing safe havens like national parks and wildlife reserves.	-	-	-	-	Government effectivenes s, characterize d by the ability to effectively implement and enforce protective policies, is crucial for covering a higher proportion of Key Biodiversity Areas with protected areas in mountain regions.	-	-	-	-
48	-	Environmental protection is a critical factor for conserving species	Social development, including poverty	Education and awareness are also	-	Healthcare services can indirectly	The effectivenes s of government	-	-	-	-

		and their habitats. This includes protecting ecosystems from pollution, habitat loss, and other forms of environmental degradation.	reduction, can positively impact the Red List Index, as access to resources and opportunities can lessen engagement in activities that harm the environment and threaten species.	essential for the conservation of species. An educated public is more likely to support conservation efforts and reduce harmful activities that threaten species.		influence the Red List Index: healthier populations are less likely to engage in activities that harm the environment and threaten species.	policies and regulations can have a significant impact on the Red List Index. Laws and regulations that protect species and their habitats are critical for their conservation.				
49	Higher economic development often correlates with lower crime rates due to factors like improved living conditions and education, but rapid development can sometimes increase crime rates through social inequality or dislocation.	-	Social development plays a significant role in reducing homicide rates. Factors such as poverty, inequality, and social exclusion can lead to higher rates of violence and crime.	Education and awareness also play a role in reducing homicide rates. Educated individuals are more likely to have better job opportunities and lead stable lives, reducing the likelihood of engaging in criminal activity.	-	Access to healthcare services can also influence homicide rates. Improved healthcare services can help identify and treat mental health issues and reduce the likelihood of violent behavior.	The effectiveness of government policies and law enforcement can have a significant impact on homicide rates. Stronger laws and better law enforcement can deter criminal activity and reduce violence.	Population density can also contribute to higher homicide rates. Higher population densities can lead to more competition for resources and increased social tensions, which can increase the likelihood	-	-	The internet can potentially reduce crime by improving communication between law enforcement and the public, aiding crime prevention and detection, but it can also facilitate crimes like

								of violence.			online harassment or cybercrime, which could sometimes contribute to real-world violence.
50	Economic development can decrease homicide rates in the 15-49 age group by increasing education and employment opportunities, but if it leads to social inequality, it could potentially raise these rates, particularly among marginalized young adults.	-	Social development is a critical factor in reducing homicide rates among young adults. Factors such as poverty, inequality, and social exclusion can lead to higher rates of violence and crime among this age group.	Education and awareness also play a role in reducing homicide rates among young adults. Educated individuals are more likely to have better job opportunities and lead stable lives, reducing the likelihood of engaging in criminal activity.	-	Access to healthcare services can also influence homicide rates among young adults. Improved healthcare services can help identify and treat mental health issues and reduce the likelihood of violent behavior.	The effectiveness of government policies and law enforcement can have a significant impact on homicide rates among young adults. Stronger laws and better law enforcement can deter criminal activity and reduce violence.	-	-	-	Internet access could potentially lower homicide rates in the 15-49 age group through education and job opportunities, but could also contribute to violence, such as online harassment or radicalization, given the group's high internet activity.

51	Economic development is a critical factor that affects the volume of remittances as a proportion of total GDP. Countries with stronger economies tend to receive more remittances from their overseas workers.	-	Social development, including poverty reduction, can positively impact remittance volume, as individuals with access to resources and opportunities are more likely to send money back to their families in their home countries.	Education and awareness can impact fixed internet broadband subscriptions, as educated individuals are more likely to understand the benefits of broadband internet and the need for high-quality internet access.	-	-		-	-	-	-
52	Economic development can also influence fixed internet broadband subscriptions. Countries with stronger economies tend to have higher rates of fixed internet broadband subscriptions.	-	-	Education and awareness can impact fixed internet broadband subscriptions, as educated individuals are more likely to understand the benefits of broadband	Technology evel is a critical factor affecting fixed internet broadband subscriptions. Countries with advanced technological infrastructure tend to have higher	-	-	Population density can also influence fixed internet broadband subscriptions. Areas with higher population densities tend to have more demand for broadband internet, as	-	-	-

				internet and the need for high-quality internet access.	rates of fixed internet broadband subscriptions.			more people are concentrated in a smaller space.			
53	Economic development can also influence the number of internet users. Countries with stronger economies tend to have higher rates of internet usage, as individuals and businesses have more resources to invest in technology.	-	-	Education and awareness significantly influence the number of internet users, as educated individuals, understanding the internet's benefits, are more likely to use it for education, work, and communication.	-	-	-	Population density can also influence the number of internet users. Areas with higher population densities tend to have more internet users, as more people are concentrated in a smaller space.	Openness to foreign direct investment can positively impact the number of internet users by improving internet infrastructure and increasing internet service availability, thereby boosting internet usage rates.	-	-
54	Higher levels of economic development often correspond to lower tariff rates as countries aim to promote international trade and market access.	Developed countries may apply higher tariffs on environmentally unfriendly products to discourage their importation and promote sustainable practices, while potentially lowering tariffs on "green" products to encourage their	-	-	-	-	Government policy and regulation effectiveness directly impacts tariff rates, with efficient and transparent policies leading to more	-	Openness to foreign direct investment influences tariff rates, with countries encouraging investment often adopting liberal trade policies,	-	The internet, by increasing transparency and public awareness about trade policies, could influence tariff rates, with public

		production and use.					consistent, predictable tariff structures, fostering trade stability and reducing tariff barriers.		including lower tariff rates, to attract foreign investors and promote economic growth.		sentiment towards protectionism potentially pressuring governments to increase tariffs, and vice versa.
55	Economic development is a crucial factor in attracting foreign direct investment. Countries with strong and growing economies, stable political environments, and favorable business conditions tend to attract higher levels of FDI.	Strong environmental protections can attract green-industry FDI, while strict regulations may deter FDI in high-impact industries due to potential compliance costs.	-	-	A country's technological advancement and innovation capabilities can impact FDI inflows, as companies seeking advanced technologies, research and development capabilities, or skilled labor may prefer investing in technologically advanced countries.	-	Governments that demonstrate stability, transparency, and efficiency in governance are more likely to attract foreign investors.	-	-	-	Countries with widespread internet access might attract FDI in tech industries or other sectors that rely heavily on digital infrastructure.

Table S7. Coastal country list.

No.	Code	Country	No.	Code	Country
1	AGO	Angola	47	IRQ	Iraq
2	ALB	Albania	48	ISR	Israel
3	ARG	Argentina	49	ITA	Italy
4	AUS	Australia	50	JAM	Jamaica
5	AZE	Azerbaijan	51	JOR	Jordan
6	BEL	Belgium	52	KAZ	Kazakhstan
7	BEN	Benin	53	KEN	Kenya
8	BGD	Bangladesh	54	KHM	Cambodia
9	BLZ	Belize	55	KOR	Republic of Korea
10	BRA	Brazil	56	LTU	Lithuania
11	BRB	Barbados	57	LVA	Latvia
12	CAN	Canada	58	MAR	Morocco
13	CHL	Chile	59	MDG	Madagascar
14	CHN	China	60	MEX	Mexico
15	CIV	Ivory Coast	61	MMR	Myanmar
16	CMR	Cameroon	62	MUS	Mauritius
17	COD	Democratic Republic of the Congo	63	MYS	Malaysia
18	COL	Colombia	64	NAM	Namibia
19	CPV	Cabo Verde	65	NGA	Nigeria
20	CRI	Costa Rica	66	NIC	Nicaragua
21	CYP	Cyprus	67	NOR	Norway
22	DEU	Germany	68	NZL	New Zealand
23	DNK	Denmark	69	OMN	Oman
24	DOM	Dominican Republic	70	PAK	Pakistan
25	DZA	Algeria	71	PAN	Panama
26	ECU	Ecuador	72	PER	Peru
27	EGY	Egypt	73	PHL	Philippines
28	ESP	Spain	74	PNG	Papua New Guinea
29	EST	Estonia	75	PRT	Portugal
30	FIN	Finland	76	QAT	Qatar
31	FJI	Fiji	77	ROU	Romania
32	FRA	France	78	RUS	Russian Federation
33	GAB	Gabon	79	SAU	Saudi Arabia
34	GBR	United Kingdom of Great Britain and Northern Ireland	80	SLV	El Salvador
35	GEO	Georgia	81	SVN	Slovenia
36	GHA	Ghana	82	SYR	Syrian Arab Republic
37	GIN	Guinea	83	TGO	Togo
38	GMB	Gambia	84	TTO	Trinidad and Tobago
39	GRC	Greece	85	TUN	Tunisia
40	GTM	Guatemala	86	TZA	United Republic of Tanzania
41	HND	Honduras	87	UKR	Ukraine
42	HRV	Croatia	88	URY	Uruguay

43	IDN	Indonesia	89	USA	United States of America
44	IND	India	90	VNM	Viet Nam
45	IRL	Ireland	91	ZAF	South Africa
46	IRN	Iran (Islamic Republic of)			

Table S8. Magnitude of transboundary interactions.

SDG indicator	SDG target	SDG indicator	Interaction Trade		Nature-caused flows	
			Synergy	Trade-off	Synergy	Trade-off
1	1.4	Proportion of population using basic drinking water services, by location (%)	7.88	0.00	6.42	0.00
2	1.4	Proportion of population using basic sanitation services, by location (%)	12.88	2.07	8.96	0.00
3	2.2	Proportion of women aged 15-49 years with anaemia, non-pregnant (%)	1.62	0.00	8.03	0.00
4	2.2	Proportion of women aged 15-49 years with anaemia, pregnant (%)	7.70	0.00	9.81	0.00
5	2.3	Cereal yield (kg per hectare)	66.40	0.00	2.84	0.00
6	3.1	Maternal mortality ratio	6.65	0.00	7.33	0.00
7	3.2	Infant mortality rate (deaths per 1,000 live births)	20.84	0.00	11.23	0.00
8	3.2	Neonatal mortality rate (deaths per 1,000 live births)	13.78	0.00	0.00	0.00
9	3.3	Tuberculosis incidence (per 100,000 population)	6.39	3.11	0.00	0.00
10	4.1	Primary education, duration (years)	0.00	0.00	0.00	0.00
11	4.2	School enrollment, preprimary (% gross)	2.96	0.00	4.20	0.00
12	4.5	School enrollment, primary (gross), gender parity index (GPI)	0.00	0.00	0.00	0.00
13	5.1	Women Business and the Law Index Score (scale 1-100)	0.00	0.00	0.00	0.00
14	5.4	Contributing family workers, female (% of female employment) (modeled ILO estimate)	6.67	0.00	9.13	0.00

15	5.5	Proportion of seats held by women in national parliaments (% of total number of seats)	0.00	0.00	0.00	0.00
16	6.2	Proportion of population practicing open defecation, by urban/rural (%)	5.34	0.00	13.09	0.00
17	6.4	Water Use Efficiency (United States dollars per cubic meter)	45.39	2.45	12.24	0.00
18	6.4	Level of water stress: freshwater withdrawal as a proportion of available freshwater resources (%)	0.16	0.70	0.00	0.00
19	6.6	Lakes and rivers permanent water area (% of total land area)	1.58	1.47	0.00	0.00
20	6.6	Lakes and rivers seasonal water area (% of total land area)	55.45	11.25	33.29	0.00
21	7.1	Proportion of population with access to electricity, by urban/rural (%)	18.17	8.31	7.52	0.00
22	7.1	Proportion of population with primary reliance on clean fuels and technology (%)	12.22	0.00	8.56	0.00
23	7.2	Renewable energy share in the total final energy consumption (%)	5.02	8.98	1.87	0.00
24	7.3	Energy intensity level of primary energy (megajoules per constant 2017 purchasing power parity GDP)	2.46	0.00	4.91	0.00
25	8.1	Number of commercial bank branches per 100,000 adults	4.53	34.23	3.66	0.00
26	8.4	Domestic material consumption per unit of GDP, by type of raw material (kilograms per constant 2015 United States dollars)	1.59	18.31	17.43	0.00
27	8.4	Domestic material consumption per capita, by type of raw material (tonnes)	6.19	3.34	0.00	6.34
28	8.5	Wage and salaried workers, total (% of total employment) (modeled ILO estimate)	8.97	2.05	4.19	0.00
29	9.2	Manufacturing value added (constant 2015 United States dollars) as a	0.00	1.17	0.00	6.74

		proportion of GDP (%)				
30	10.4	Labour share of GDP (%)	0.00	1.07	0.00	0.00
31	10.7	Number of refugees per 100,000 population, by country of origin (per 100,000 population)	0.00	0.00	0.00	0.00
32	11.1	Urban population (% of total population)	4.09	1.36	0.00	0.00
33	11.1	Urban population growth (annual %)	8.22	0.00	0.00	0.00
34	11.6	Annual mean levels of fine particulate matter (population-weighted), by location (micrograms per cubic meter)	23.53	0.00	0.00	0.00
35	11.6	PM2.5 air pollution, population exposed to levels exceeding WHO guideline value (% of total)	21.20	2.30	31.76	0.00
36	11.6	PM2.5 air pollution, mean annual exposure (micrograms per cubic meter)	11.57	4.03	20.02	5.34
37	12.2	Total natural resources rents (% of GDP)	41.20	9.28	16.23	0.00
38	12.2	Domestic material consumption per unit of GDP, by type of raw material (kilograms per constant 2015 United States dollars)	1.59	18.30	17.31	0.00
39	12.2	Domestic material consumption per capita, by type of raw material (tonnes)	5.30	3.14	0.00	5.79
40	13.1	Total number of people affected by floods per 100,000	14.43	15.57	0.19	0.00
41	13.1	Energy-related CO2 emissions per capita (tCO2/capita)	0.00	0.00	0.00	0.00
42	14.4	Anual growth rate of capture fisheries production	20.64	12.40	0.00	0.00
43	14.4	Annual growth rate of aquaculture production (of total %)	13.12	0.00	0.00	0.00
44	14.4	Annual growth rate of total fisheries production (metric tons)	0.00	10.36	5.53	0.00

45	15.1	Forest area (% of land area)	0.00	0.63	0.00	0.95
46	15.1	Average proportion of Terrestrial Key Biodiversity Areas (KBAs) covered by protected areas (%)	12.68	0.00	6.01	0.00
47	15.4	Average proportion of Mountain Key Biodiversity Areas (KBAs) covered by protected areas (%)	2.15	0.00	0.00	0.00
48	15.5	Red List Index	0.00	2.19	1.78	2.87
49	16.1	Annual number of deaths from homicide per 100,000 people	0.00	0.00	0.00	0.00
50	16.1	homicide rate by age (15-49)	0.00	0.00	0.00	0.00
51	17.3	Volume of remittances (in United States dollars) as a proportion of total GDP (%)	0.00	0.00	0.00	0.00
52	17.6	Fixed Internet broadband subscriptions per 100 inhabitants, by speed (per 100 inhabitants)	0.00	0.00	0.00	0.00
53	17.8	Internet users per 100 inhabitants	0.00	0.00	0.00	0.00
54	17.12	Average tariff applied by developed countries, most-favored nation status, by type of product (%)	0.00	0.00	0.00	0.00
55	17.13	Foreign direct investment, net inflows, as a proportion of GDP (%)	0.00	0.00	0.00	0.00
		Sum	500.54	178.05	273.56	28.03
		Average	9.10	3.24	4.97	0.51
